# A Novel CT Imaging System with Adjacent Double X-Ray Sources

**DOI:** 10.1155/2013/391212

**Published:** 2013-11-14

**Authors:** Mou An, Yaoqin Xie

**Affiliations:** Key Laboratory of Health Informatics, Shenzhen Institutes of Advanced Technology, Chinese Academy of Sciences, 1068 Xueyuan Avenue, Shenzhen 518055, China

## Abstract

Current computed tomography (CT) scanners rotate fast to reduce motion artifact. X-ray tube must work in a high power to make the image clear under short exposure time. However, the life span of such a tube may be shortened. In this paper, we propose a novel double sources CT imaging system, which puts two of the same X-ray sources closely with each other. The system is different from current dual source CT with orthogonal X-ray sources. In our system, each projection is taken twice by these two sources to enhance the exposure value and then recovered to a single source projection for image reconstruction. The proposed system can work like normal single source CT system, while halving down the working power for each tube.

## 1. Introduction

Current computed tomography (CT) scanners acquire multiple projection images (~1000 frames) [1, 2]. A more powerful X-ray tube could emit more X-ray photons during one exposure frame and then cost less time to ensure constant exposure dose. Twice the power gives the possibility of increasing the scanning speed. However, the cathode filament electric current must be doubled to ensure emitting twice the number of electrons, which is harmful to the lifetime of the cathode filament and the positive plate. Zhang et al. [3–6] proposed a multiplexing radiography technology based on carbon nanotube field emission, which is an effective method to gather X-ray radiation from more than one tube to enhance the exposure rate in one frame. In this case, the X-ray sources must be modulated easily. Otherwise, the maximum tube voltage of these sources is limited by current technology.

In this paper, a novel CT imaging system is developed by placing two of the same X-ray tubes close to each other, along with a common detector. The system is different from current dual source CT system, where two sets of tube detectors are placed orthogonally [7–9]. In this system, the detector detects X-rays coming from two sources simultaneously. Two sources working together can make the exposure rate the same as a single source with half the exposure time. 

In the following sections, first we describe the structure of adjacent double X-ray sources and then propose a method to separate the overlapped projections for image reconstruction. For demonstration, fan-beam data acquisition and image reconstruction are presented, but the method can be easily extended to cone beam image reconstruction. Lastly, we discuss the potential benefits and limitations of the proposed method.

## 2. Materials and Methods

### 2.1. CT System with Adjacent Double X-Ray Sources

**Figure 1:**
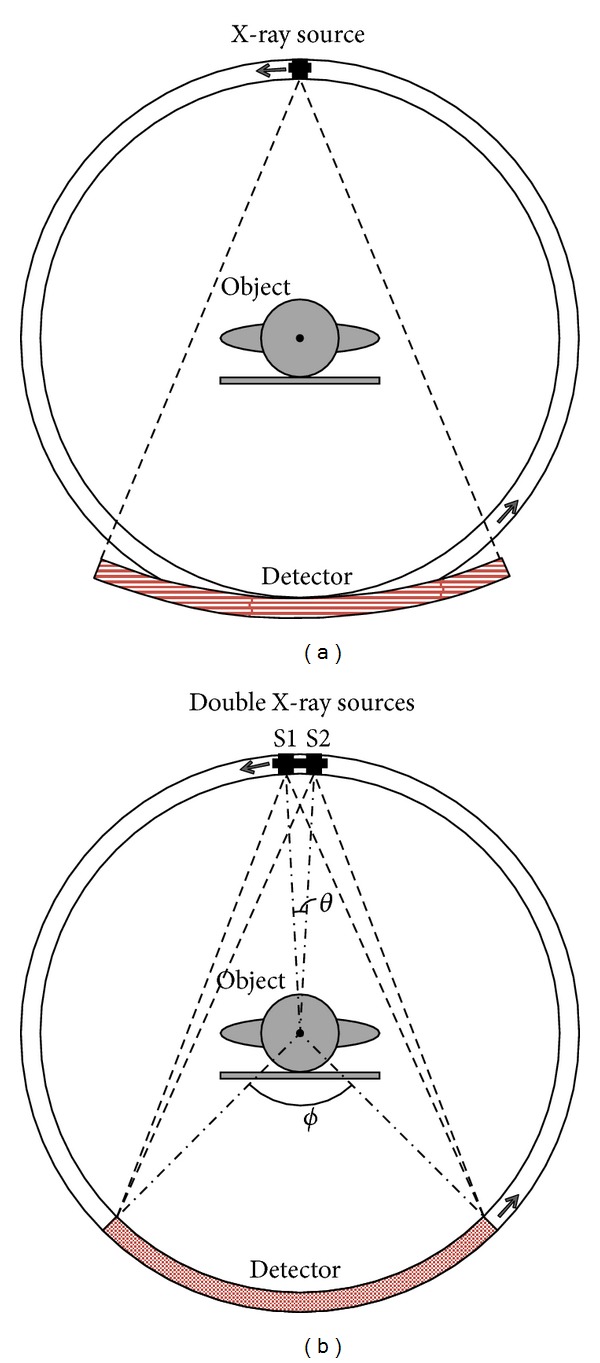
(a) Current CT scanner system using a single-beam X-ray source and arc detector, whose focus is located in the source. (b) The proposed adjacent double X-ray sources system, in which the focus of the arc detector is located in the rotating center.

The structure of traditional CT scanner system is illustrated in Figure 1(a). It can be seen that one X-ray source located in the focal point of the arc detector and synchronously rotates in anticlockwise direction along with the arc detector [10–12]. The rotation axis is the midpoint of the radius of curvature.  Figure 1(b) gives us the proposed adjacent double X-ray sources system structure. In this system, one arc detector faces two X-ray sources. Different from Figure 1(a), there are two of the same X-ray sources that synchronously rotate with the arc detector. The rotation axis is no more the midpoint of the radius of curvature but the center of the arc detector.

In this illustration about proposed double X-ray sources scanning system, sources are labeled as S1 and S2. As shown in Figure 1(b), **θ** is the angle between two sources relative to the rotation center. Two sources synchronously rotate in anticlockwise direction along with the arc detector. If we assume that the rotation speed is **ω** and the time between two consecutive sampling events is *δt*, then *θ* = *ω* · *δt*. In the processing of projections acquisition, the position of S1 at one sampling event will be replaced by S2 in the next sampling event. And, as shown in Figure 1(b), the object must be covered by X-rays coming from both of the two sources. Both sources are turned on during the whole scanning procedure.

### 2.2. Projection Separating

Just like the Hadamard multiplexing radiography (HMR) method [5], we assume the *N*th projection under two sources has the form *y*_*N*_ and the *N*th projection under one source has the form *x*_*N*_. The total projections number is *M*. Let *X* = [*x*_1_,…, *x*_*N*_,…, *x*_*M*_]^*T*^ be the original projections serials; the overlapped projections serials *Y* = [*y*_1_,…, *y*_*N*_,…, *y*_*M*_]^*T*^ are related to the original serials by a linear transform:

(1) $$\begin{array}{c} \;Y=SX. \end{array}$$

The *S*-matrices consist of only 1 and 0, which correspond to the existence or not of the single source. The definite form is as follows:

(2) $$\begin{array}{c} S=\left[\begin{array}{cccc} 1 & 1 & 0 & \cdots \\ 0 & 1 & 1 & \ddots \\ \ddots & \ddots & \ddots & \ddots \\ 1 & 0 & 0 & 1 \end{array}\right], \end{array}$$

where the row number of matrix means the acquisition sequence number in one scanning circle. And the column number of each row means the position of the sources.

As an example for the *S* matrix of order *M* = 4, the convolution process that occurs in the experiment can be expressed succinctly in a matrix notation as

(3) $$\begin{array}{c} \left[\begin{array}{c} y_{1}\\ y_{2}\\ y_{3}\\ y_{4} \end{array}\right]=\left[\begin{array}{cccc} 1 & 1 & 0 & 0\\ 0 & 1 & 1 & 0\\ 0 & 0 & 1 & 1\\ 1 & 0 & 0 & 1 \end{array}\right]\left[\begin{array}{c} x_{1}\\ x_{2}\\ x_{3}\\ x_{4} \end{array}\right]=\left[\begin{array}{c} x_{1}+x_{2}\\ x_{2}+x_{3}\\ x_{3}+x_{4}\\ x_{4}+x_{1} \end{array}\right]. \end{array}$$

**Figure 2:**
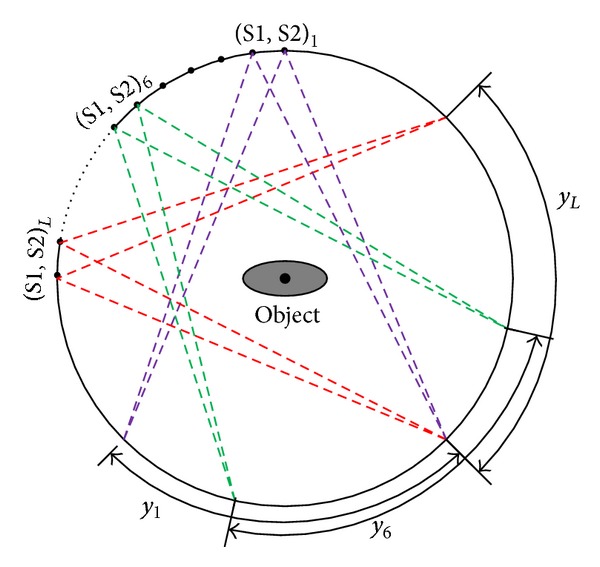
Rotation of the adjacent double X-ray sources system. (S1, S2)_*N*_ represents the two sources at the *N*th projection angle in one scanning circle. After the sources detector pairs rotate *L* × *θ* degrees, the *L*th projection *y*_*L*_ is completely separated from the first projection *y*_1_.

The number of rows of the S-matrices equals *M*, that is, the projections number, and must meet the condition *θ* · *M* = 360°. For a big enough *M*, the arc angle of detector *ϕ* satisfies the relationship *L* · *θ* ≤ *ϕ* < (*L* + 1) · *θ*, which means that after taking *L* projections, the current position of projections received by the rotated detector is completely separated from that received on the original position, as shown in Figure 2. 

**Figure 3:**
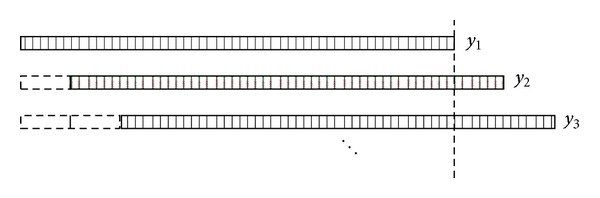
Overlapped projections with the drifted detector. In two adjacent overlapped projections, there exist two original projected signals that come from the same angle source and that are accepted by the drifted detector.

 For simplicity and clarity, in Figure 3, we changed the arc detector to flat. In this illustration, we assume that *y*_1_ = *x*_1_ + *x*_2_, *y*_2_ = *x*_2_′ + *x*_3_, *y*_3_ = *x*_3_′ + *x*_4_,…, and *x*_2_ and *x*_2_′ are the projections about the same angle but the detector drifts. The vertical dashed line in Figure 3 cuts *y*_2_ into two parts. It is required that the object must be covered by X-rays coming from both of the two sources; as a result, the right side of *x*_2_′ in *y*_2_ is zero. On the other hand, the extension line part of *x*_2_′ in *y*_2_ is zero too. The positions of the projected images in *x*_2_ and *x*_2_′ totally coincide. As a result, it can be absolutely expressed by *y*_2_ = *x*_2_ + *x*_3_; then we can subtract *x*_2_ just by *y*_1_ − *y*_2_ = *x*_1_ − *x*_3_. In the same way, *x*_3_′ in *y*_3_ equals *x*_3_; we can also subtract *x*_3_ just by *x*_3_ − *y*_3_ = −*x*_4_. We do this thing until the projections *y*_*L*_ drift totally outside the vertical dashed line; then we can recover *x*_1_. The general expression is performed as

(4) xi=yi−yi+1+yi+2−⋯+(−1)Lyi+L=∑n=0L(−1)nyi+n.

Here, if *i* + *n* > *M*, *y*_*i*+*n*_ means the sources rotate more than one circle and equals *y*_*i*+*n*−*M*_. *L* means that after the sources rotate *L* × *θ* degrees, the projection *y*_*i*+*L*_ is totally separated from the original projection *y*_*i*_.

### 2.3. Image Reconstruction

**Figure 4:**
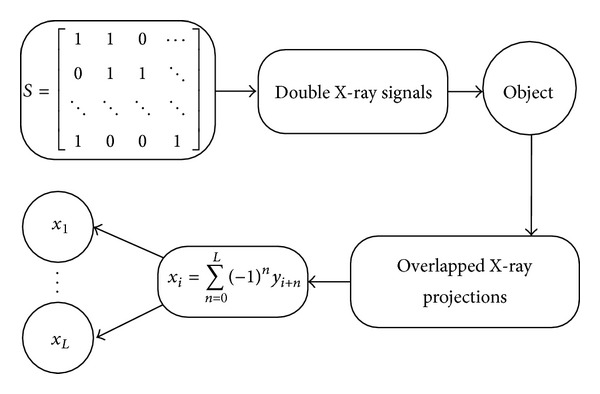
Flow chart of the imaging procedure of the adjacent double X-ray sources system. The incident X-ray comes from two sources transmitted through the object and formed an overlapped X-ray projection recorded by the arc X-ray detector. After a complete set of overlapped projections were acquired, the proposed algorithm was applied to the projection data to recover the original projection images.

The flow chart of the general imaging and data processing procedure for this adjacent double X-ray sources system is shown in Figure 4. After the overlapped projections are separated, the traditional fan-beam reconstruction algorithm is applied for image reconstruction [13–15].

By means of variable substitution, we get the FBP method for the fan-beam reconstruction [15]

**Figure 5:**
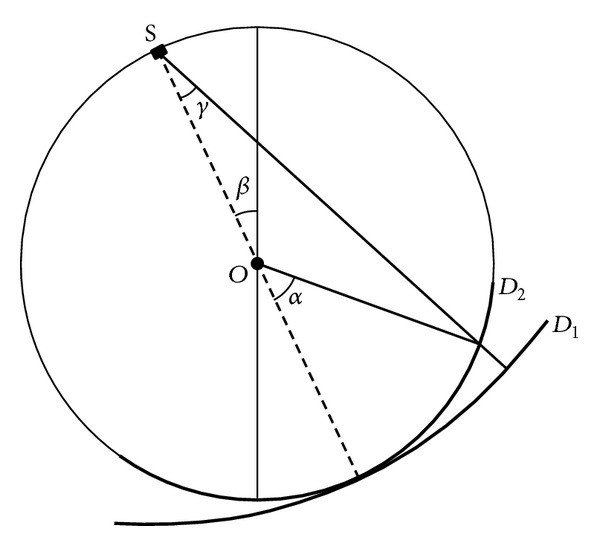
Illustration of the two kinds of arc detectors. *S* is the center of detector *D*_1_ and *O* is the center of detector *D*_2_.

(5) f(r,φ) =∫02π1D′2∫−π/2π/2(cos⁡γ)g(γ,β)hfan(γ′−γ)dγdβ,

where *h*_fan_(*γ*) = (*D*/2)(*γ*/(sin*γ*))^2^ *h*(*γ*) and g(*γ*, *β*) is the projection accepted by arc detector D1 on the rotated degree *β*, as illustrated in Figure 5. The opening angle relative to the source S between X-ray passed through the reconstructed point *P* and the detector's central line (dotted line) is *γ*. If we replace the detector D1 by D2, the received image by D2 is *g*′(*α*, *β*) and *g*(*γ*, *β*) = *g*′(*α*, *β*). Then the FBP method (5) can be rewritten as

(6) $$\begin{array}{c} f\left(r,\varphi \right)=\int_{0}^{2\pi }\frac{1}{{D^{\prime 2}}^{}}\int_{-\pi }^{\pi }\left(\cos\frac{\alpha }{2}\right)g^{\prime }\left(\alpha ,\beta \right)h_{\text{fan}}^{\prime }\left(\alpha^{\prime }-\alpha \right)d\alpha d\beta , \end{array}$$

where *h*_fan_′(*α*) = (*D*/4)(*α*/(sin(*α*/2)))^2^ *h*(*α*).

## 3. Results and Discussion

**Figure 6:**
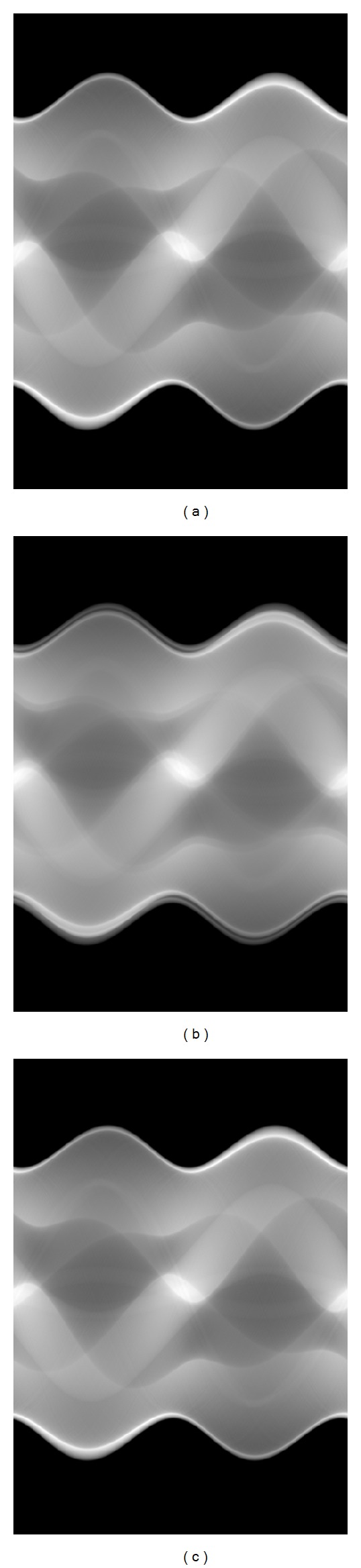
Sinogram of projections: from left to right it is under single X-ray source, overlapped and separated under double X-ray sources.

To demonstrate the feasibility of the proposed system, the Shepp-Logan phantom was applied to simulate double sources exposure. The opening angle of the two sources to the rotation center was 1°. The radius of the circular orbit was 60 cm; that is, *D* = 60 cm. The maximum fan-subtending angle was *ϕ* = 32°. The projections were evenly acquired in [0°, 360°] with a sampling angle of *θ* = 2*π*/360, and *ϕ* was equally sampled in [−16°, 16°] with a total of 512 X-ray sums.  Figure 6 gives the noise-free sinogram of the projections under one X-ray source, overlapped and separated under double X-ray sources. Because neither of the two X-ray sources was on the central line of the arc detector, after being separated, the projections (c) were a little vertically drifted compared with (a). During the reconstruction, this should be taken into account.

**Figure 7:**
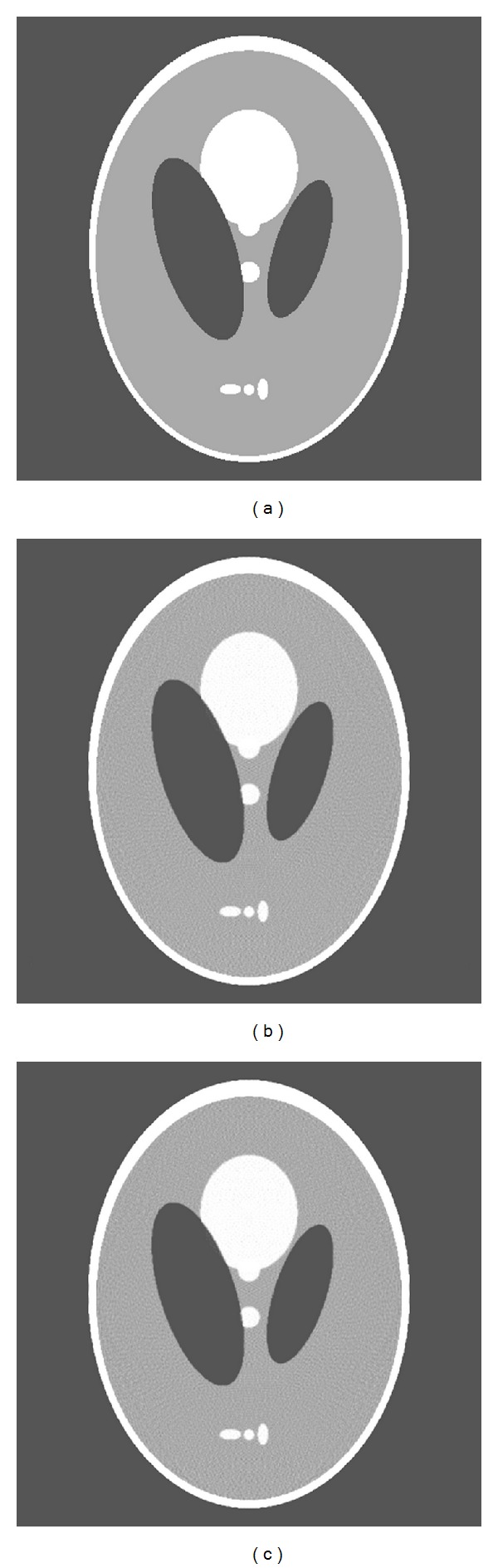
(a) Shepp-Logan phantom. (b) Reconstructed image from the projections under single X-ray source. (c) Reconstructed image from the signals separated from the overlapped projections under two X-ray sources. Display window: [0.1 0.3].

**Figure 8:**
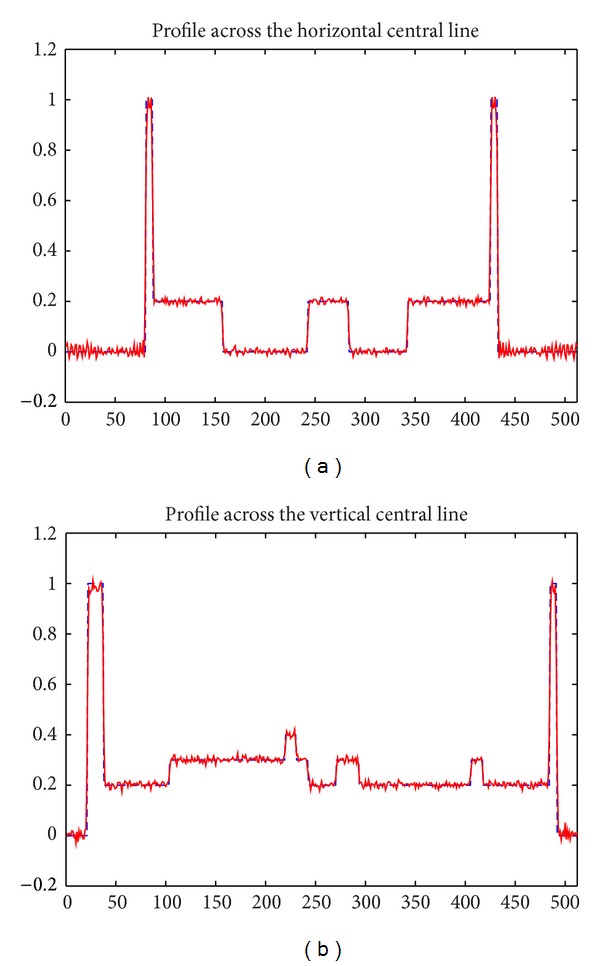
Profile comparison between reconstructed (solid red line) and true image (dashed blue line) on the horizontal and vertical central line.

The Shepp-Logan phantom is shown in Figure 7(a). for comparison, the reconstructed images using (5) for single X-ray source and using (6) for double X-ray sources are shown in Figures 7(b) and 7(c). Each of them is 512 × 512 pixels and displayed based on the conventional greyscale window setup of [0.1, 0.3] [16]. The image reconstructed from the overlapped projections seems to have the same quality as the image reconstructed from the projections under single X-ray source. Mean square error (MSE) of Figure 7(b) is 0.039 and for Figure 7(c) is 0.040, respectively. Quantitative agreement between the reconstructed image in Figure 7(c) and the true phantom in Figure 7(a) can be seen in Figure 8.

In the simulation above, we present preliminary studies that demonstrate image reconstruction noise-free with the overlapped projections under adjacent double X-ray sources. In this imaging system, two X-ray sources must be the same, that is, the machine model, radiation field of photons. In practice, radiation field could be adjusted by the working voltage and current. Our results are provided about the fan-beam reconstruction, but the method is suitable for cone beam CT; just make sure that the two adjacent X-ray sources are placed parallel to the rotating direction and that the opening angle of the two sources to rotation center agree with the acquisition angle. In normal CT scanning, we take hundreds of projections during one circle. The angle rotated between two sampling is so small that two X-ray sources must be placed close to each other. We could place two identical cathode filament in one X-ray tube. However, sparse projections reconstruction acquires fewer projections than conventional methods; the sampling angle is relatively big, which facilitates placing two X-ray tubes together.

As compared to traditional CT system, the proposed adjacent double X-ray sources system can squeeze double X-ray photons during one unit exposure time; thus, it can easily enhance the illumination for some situations that need a high exposure rate such as fat patients. Also, high exposure rate during one unit exposure time gives the ability to halve down the projections acquisition time to reduce the motion artifact. On the other hand, if fast exposure is not required, two sources that work in a half power can give enough X-ray photons, which can prolong the lifetime of the tubes. 

**Figure 9:**
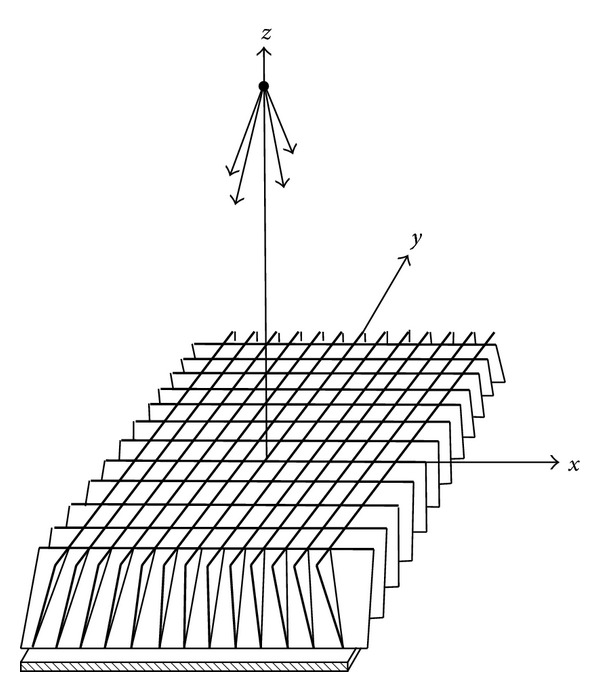
Antiscatter collimators.

The main advantage of such a configuration is the power, since the two tubes can operate simultaneously. However, several issues should be tackled before it can be used in clinic. First, scatter is a big issue that can influence the quality of the reconstructed images.  Figure 9 is the schematic diagram of the antiscatter collimators. Each lead inclined to one focus, that is, the source. In our proposed system, two sources are placed in cross-section perpendicular to the rotating axis and parallel to the rotating direction. This kind of antiscatter collimators could not be used. But we can take out the leads on one direction; for example, we take out the leads parallel to *y*-axis. Then the rest of the lead inclined to line across the two sources. This is a compromised way to reduce part of the scatter. Another big issue is the CNR after the preprocessing step in (4). According to the result in [5], the separated projections under three sources are still clear enough. 

## 4. Conclusions

In this paper, we developed a novel structure of CT system with adjacent double X-ray sources. In the proposed system, two X-ray sources work simultaneously. Therefore, the tube can work longer under the same exposure dose as traditional single source or it can give a high exposure rate for some situations such as fat patients. Because the sources are not necessary to be modulated and the reconstruction method is based on the FBP algorithm, the system can be realized by current X-ray tube technology and CT structure.
